# Multilayered Molybdate Microflowers Fabricated by One‐Pot Reaction for Efficient Water Splitting

**DOI:** 10.1002/advs.202206952

**Published:** 2023-03-22

**Authors:** Jingyi Wang, Jianrui Feng, Yuying Li, Feili Lai, Gui‐Chang Wang, Tianxi Liu, Jiajia Huang, Guanjie He

**Affiliations:** ^1^ School of Chemical Engineering Zhengzhou University Zhengzhou 450001 P. R. China; ^2^ Department of Chemical Engineering University College London London WC1E 6 EB UK; ^3^ Department of Chemistry KU Leuven Celestijnenlaan 200F Leuven 3001 Belgium; ^4^ Key Laboratory of Advanced Energy Materials Chemistry (Ministry of Education) and the Tianjin Key Lab and Molecule‐Based Material Chemistry College of Chemistry Nankai University Tianjin 300071 China; ^5^ Key Laboratory of Synthetic and Biological Colloids Ministry of Education School of Chemical and Material Engineering International Joint Research Laboratory for Nano Energy Composites Jiangnan University Wuxi 214122 P. R. China

**Keywords:** bifunctional electrocatalyst, doping, lanthanum ammonium molybdate, lattice defects, microflowers, water splitting

## Abstract

The development of high‐performance, low‐cost and rapid‐production bifunctional electrocatalysts towards overall water splitting still poses huge challenges. Herein, the authors utilize a facile hydrothermal method to synthesize a novel structure of Co‐doped ammonium lanthanum molybdate on Ni foams (Co‐ALMO@NF) as self‐supported electrocatalysts. Owing to large active surfaces, lattice defect and conductive channel for rapid charge transport, Co‐ALMO@NF exhibits good electrocatalytic performances which requires only 349/341 mV to achieve a high current density of 600 mA cm^−2^ for hydrogen evolution reaction (HER) and oxygen evolution reaction (OER), respectively. Besides, a low cell voltage of 1.52 V is required to reach the current density of 10 mA cm^−2^ in alkaline medium along with an excellent long‐term stability for two‐electrode configurations. Density functional theory calculations are performed to reveal the reaction mechanism on Co‐ALMO@NF, which shows that the Mo site is the most favorable ones for HER, while the introduction of Co is beneficial to reduce the adsorption intensity on the surface of Co‐ALMO@NF, thus accelerating OER process. This work highlighted the importance of the structural design for self‐supporting electrocatalysts.

## Graphical abstract

Co‐doped ammonium lanthanum molybdate compounds are synthesized as bifunctional self‐supporting electrocatalysts by facile hydrothermal method. The lattice defects induced by metal doping excite more active sites and enhance electronic conductivity. Consequently, the cell voltage of the constructed two‐electrode system is only 1.52 and 1.64 V to drive the current densities of 10 and 100 mA cm^−2^.

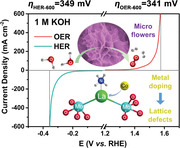

## 1 Introduction

Due to the inexhaustible supply of water, electrochemical water splitting has obtained considerable attention as a sustainable and green way to generate hydrogen and oxygen.^[^ ^1^, ^2^, ^3^ ^]^ Noble metal‐based materials are considered as the most efficient electrocatalysts for the water splitting at present,^[^ ^4^ ^]^ but the paucity and high cost are significant problems. In addition, existing non‐noble metal electrocatalysts are difficult to meet the demands of effective, stable performances, and low‐cost simultaneously, which limits the wide‐scale availability of the water electrolysis.^[^ ^5^ ^]^

The research on bifunctional electrocatalysts which hold dual electrocatalytic capabilities for hydrogen evolution reaction (HER) and oxygen evolution reaction (OER) have promoted the development and application of the overall water splitting.^[^ ^6^, ^7^, ^8^ ^]^ The application of bifunctional electrocatalyst avoids the problem of electrolyte incompatibility and further simplifies the device and reduces the costs of water electrolysis.^[^ ^9^ ^]^ A variety of strategies have been applied to develop transition metal‐based bifunctional electrocatalysts for water electrolysis^[^ ^10^ ^]^ and following strategies are effective for developing bifunctional electrocatalysts with high activity and stability: 1) more accessible active sites can be exposed via subtly designing the microtopography for instance nanorod, core–shell, or 3D porous structure. Among them, larger specific area is more easily obtained by flower‐like structure due to the unique morphology. Meng et al. explored a 3D flower‐like WP_2_ nanowire arrays electrocatalyst in situ grown on nickel foam, which achieved 10 mA cm^−2^ at a small cell voltage of 1.65 V for overall water splitting since abundant active sites and the shorten transfer path of electrons.^[^ ^11^ ^]^ 2) In particular, the doping of active species can be realized concurrently in the preparation progress, thus further enhancing the catalytic activity. Metal doping is one of the methods to induce defects in nanocatalytic materials, the electronic structure and the coordination environment can be adjusted by doping with other metal elements, which increase the catalytic active sites and reduce the energy barrier of the hydrolysis reaction, which can increase the intrinsic activity and stability of catalysts thus exhibiting excellent electrocatalytic activity and stability.^[^ ^12^, ^13^ ^]^ 3) Rare‐earth elements are used to provide additional tunability of materials including excellent temperature indicators^[^ ^14^, ^15^ ^]^ and controllable pore environments due to the unique properties of 4f electrons and lattice defects modification.^[^ ^16^, ^17^ ^]^ Das and co‐workers synthesized La‐doped copper oxide nanoparticles by the coprecipitation and drop‐casting technique, Cu_1−_ *_x_* La*_x_* O required a cell voltage of 1.6 V to achieve the current density of 10 mA cm^−2^ in an alkaline solution.^[^ ^18^ ^]^

Meanwhile, the method of mixing materials with binders may cause additional resistance, unsuitable interface effects of as‐prepared electrodes which are adverse for ion and electron transport during the electrocatalytic process, leading to a reduced activity.^[^ ^19^, ^20^ ^]^ Hybridizing a conductive substrate with a electrocatalyst is a direct and effective way to improve overall performances. The tight combination of active species and substrates ensures the high structural stability of the electrocatalyst, which also reduces the adhesion of bubbles on the surface of electrodes, which is beneficial to promoting electrocatalytic reactions. The advantages of 1D materials to encourage the axial transfer of electrons and the advantages of 2D materials to increase the specific surface area can form a 3D structure that is interconnected and rich in pores.^[^ ^21^, ^22^ ^]^ In particular, Ni foam has been proven to be an effective conductive substrate in a strong alkaline medium, with good corrosion resistance, high porosity, good conductivity and superb mechanical strength, so as to be used as stable skeleton to grow active materials directly.^[^ ^23^, ^24^ ^]^ For example, Huang et al. formed an array of 3D Mo‐doped CoSe_2_ nanosheets on Ni foams by a three‐step process containing electrodeposition, hydrothermal and selenylation, which acquired a cell voltage of 1.54 V to reach the current density of 10 mA cm^−2^ under an alkaline condition.^[^ ^25^ ^]^

Mo‐based compounds have attracted increasing research interests as a new type of water splitting electrocatalysts recently. A series of molybdates, MMoO*_x_* (M = Co, Ni, Fe, Cu, Zn, Mn), have been proved to be excellent candidates as active electrocatalysts.^[^ ^26^, ^27^, ^28^ ^]^ On account of the superior corrosion resistance and electrochemical redox activities, MMoO*_x_* is usually dynamic in electrocatalytic systems and can stably exists in basic media.^[^ ^27^, ^29^, ^30^ ^]^ However, MMoO*_x_* nanostructures also possess the shortcoming of easy agglomeration. There are rare researches on the preparation of highly distributed MMoO*_x_* delivering superior activity and stability with ordered nano‐ or micromorphology by a facile preparation method.^[^ ^31^, ^32^ ^]^

Herein, we synthesize Co doped‐ammonium lanthanum molybdate microflowers on Ni foams (denoted as Co‐ALMO@NF) through a single‐step hydrothermal method as a bifunctional electrocatalyst for water splitting. The microflowers composed of nanosheets uniformly disperse on the Ni foam, and the porous structure provides a large accessible surface area for the electrolyte, which makes for the rapid occurrence of the electrocatalytic reaction. To further expose the reaction mechanism of HER and OER process, density functional theory (DFT) calculations were performed. The result show that the Mo site is the most promising site for HER process, while the introduction of Co is beneficial to reduce the adsorption intensity on the surface of Co‐ALMO@NF, thus accelerating the OER development. Therefore, Co‐ALMO@NF displays more active edges and shows superior electrocatalytic activity for overall water splitting, which delivers a current density of 600 mA cm^−2^ at an overpotential of 349 mV for HER and 341 mV for OER in 1.0 M KOH. Moreover, Co‐ALMO@NF only requires a cell voltage of 1.52 V to reach the current density of 10 mA cm^−2^. The innovative structural design in this work provides a facile methodology for the development of efficient bifunctional electrocatalysts.

## 2 Results and Discussion

**Figure 1:**
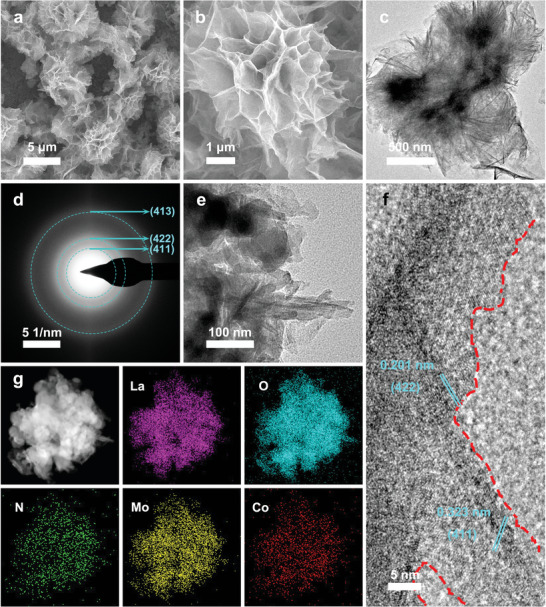
a,b) SEM images of Co‐ALMO@NF under different magnifications. c) TEM image, d) SAED pattern, and e,f) HRTEM images of Co‐ALMO@NF. g) STEM image and corresponding elemental mapping images of N, La, Mo, O, and Co for Co‐ALMO@NF.

The one‐step hydrothermal approach was applied to synthesize Co‐ALMO@NF microflowers, the specific procedures were described in experimental details. Briefly, certain amounts of metal salts (La(NO_3_)_3_·6H_2_O, (NH_4_)_6_Mo_7_O_24_·4H_2_O, and (CH_3_COO)_2_Co·4H_2_O), fumaric acid, and urea, were dispersed in Deionized water (DI water) in an autoclave, and the pretreated Ni foam was immersed into the solution. After 9 h reaction time, the self‐supported Co‐ALMO@NF electrocatalyst was obtained and directly used as an electrode. We first examined the surface morphology of as‐prepared electrocatalysts by scanning electron microscopy (SEM). As shown in **Figure** **1**a, 3D micro flowers with a size of ≈5 µm are dominant and evenly grown on the Ni foam skeleton. The high‐magnification SEM image (Figure 1b) reveals that thin and curved nanosheets radiate from the center to form the hierarchical 3D spherical microflower, which greatly enhance the active surface area of the Co‐ALMO@NF compared to the 2D materials with large layered structures, while enabling lower contact resistance for further accelerate the electron transport across the electrode/electrolyte interface. The transmission electron microscopy (TEM) image of Co‐ALMO@NF in Figure 1c is consistent with the SEM observation, exhibits the morphology of micro flower. The lamellas of Co‐ALMO@NF are loosely connected and the gap between them is conducive to the expansion of the surface area, which is also testified by nitrogen adsorption–desorption measurement (Figure S1, Supporting Information). The coating of the micrometer flowers resulting in a much larger specific surface area for Co‐ALMO@NF (58.96 m^2^ g^−1^) than that for ALMO@NF (32.61 m^2^ g^−1^) and pure Ni foam (10.28 m^2^ g^−1^), which contribute to faster reaction kinetics with higher surface reactivity. The selected area electron diffraction (SAED) analysis (Figure 1d) reveals derived rings from the (413), (422), and (411) planes of NH_4_La(MoO_4_)_2_. The high‐resolution TEM (HRTEM) image in Figure 1e illustrates the morphology of stacked nanosheets, and Figure 1f shows abundant crystalline‐noncrystalline boundaries, which excite more electrocatalytically active sites.^[^ ^33^ ^]^ The distinct lattice spacing of 0.201 and 0.323 nm in the crystalline part are ascribed to the (422) and (411) plane of NH_4_La(MoO_4_)_2_, respectively. Lattice defects are observed at the edge of nanocrystalline, which is because replacing La^3+^ with Co^2+^ will result in defect oxygen near the Co dopant. Long‐range disordered and short‐range ordered structural features in the amorphous phase can effectively increase surface defects and unsaturated coordination sites.^[^ ^34^ ^]^ The scanning TEM (STEM) and corresponding energy dispersive X‐ray spectroscopy elemental mapping images (Figure 1g) further prove the existence and uniform distribution of elemental N, La, Mo, O, and Co in Co‐ALMO@NF.

**Figure 2:**
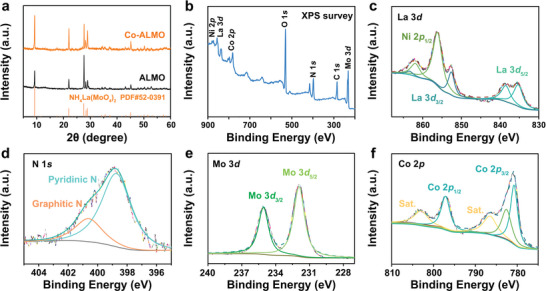
a) XRD patterns, b) XPS survey, c) La 3d, d) N 1s, e) Mo 3d, and f) Co 2p spectra for Co‐ALMO@NF.

The crystalline phase structure of Co‐ALMO is further characterized by X‐ray diffraction (XRD). As shown in **Figure** **2**a, the dominating diffraction peaks of Co‐ALMO approximated at 9.2°, 21.9°, 27.6°, 28.4°, 28.9°, and 45.1° belong to the (200), (211), (411), (420), (221), and (422) crystal planes of NH_4_La(MoO_4_)_2_ Powder Diffraction File (PDF No. 52‐0391). The XRD patterns of Co‐ALMO and ALMO are basically the same, indicating that the doping of Co did not change the basic crystalline phase of NH_4_La(MoO_4_)_2_, but it is noteworthy that the three main peaks of Co‐ALMO show a slight rightward shift compared with that of ALMO (Figure S2, Supporting Information), representing that the introduction of Co causes the cell shrinkage, which proves that Co (ionic radius: 0.065 nm) is doped into the cell in the form of substituted La (ionic radius: 0.106 nm). Compared with XRD standard data, the intensity of the peak at 27.6° increases obviously, indicating that (411) crystal plane of NH_4_La(MoO_4_)_2_ is preferential crystallographic orientation. The X‐ray photoelectron spectroscopy (XPS) is conducted to attain the valence state and elemental compositions of Co‐ALMO@NF. As depicted in Figure 2b, the XPS survey spectrum confirms the existence of Ni, La, Co O, N, and Mo elements. The high‐resolution XPS spectrum of La 3d (Figure 2c) shows two peaks located at 835.4 and 838.6 eV, which correspond to La 3d_5/2_ and mainly in the form of La^3+^,^[^ ^35^, ^36^, ^37^ ^]^ two small peaks at 852.6 and 863.4 eV can be classified to La 3d_3/2_. La 3d and Ni 2p signals partially overlap, peaks located at 856.3 and 862.1 eV correspond to Ni 2p_1/2_. The high‐resolution XPS spectrum of N 1*s* (Figure 2d) can be deconvolved into two major peak types, which suggests the existence of pyridinic N and graphitic N, respectively.^[^ ^38^ ^]^ In the XPS spectrum of Mo 3d, two major peaks correspond to Mo 3d_5/2_ and Mo 3d_3/2_, respectively (Figure 2e),^[^ ^39^ ^]^ attributing to Mo^6+^. In accordance with the previous report, these peaks of Mo mainly belong to the molybdate compounds, thus further providing supports for the existence of molybdate in Co‐ALMO@NF.^[^ ^40^, ^41^ ^]^ In Figure 2f, the XPS spectrum of Co 2p displays a core level peak curve which is fitted to two spin–orbit doublets and three shake‐up satellites. The two peaks at 780.8 and 797.1 eV are attributed to Co 2p_3/2_ and Co 2p_1/2_, respectively.^[^ ^42^, ^43^ ^]^ The satellite peaks at 782.6, 786.6, and 803.1 eV are due to oxidized Co species.^[^ ^44^, ^45^ ^]^ Based on previous research,^[^ ^46^ ^]^ the higher Mo oxidation state of Co‐ALMO@NF maybe due to more electron transfer at the lattice defects and electrons transfer from Mo atoms to the adjacent atoms.^[^ ^47^ ^]^ Meanwhile, the higher binding energy in Co 2p signifying more additional charges at the side of Co atoms.^[^ ^48^ ^]^ Besides, the peaks of O 1*s* concentrated at 530.6, 531.3, and 532.2 eV can be indexed to Mo‐O, defect oxygen and adsorbed H_2_O on Co‐ALMO, respectively (Figure S3, Supporting Information). The presence of defective oxygen supports the defects in the lattice in the electron microscopy spectrum.^[^ ^49^ ^]^ As shown in Figure S4a in the Supporting Information, the presence of Co can be clearly observed on the XPS survey spectra, which proves the successful introduction of Co. Compared to the Mo 3d and La 3d spectra of ALMO@NF, the Co‐ALMO@NF possesses slightly higher binding energy than pristine ALMO@NF, illustrating the electron transfer from ALMO to Co caused by the addition of Co. The difference in electron density due to electron transfer can be regarded as an origin for improved active sites of the bifunctional electrocatalyst.

**Figure 3:**
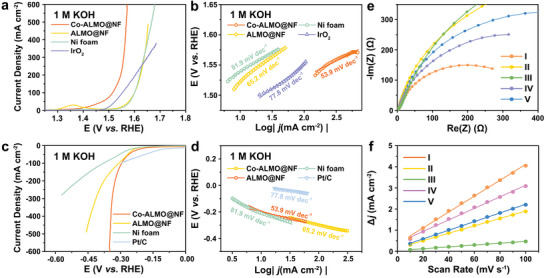
a) Polarization curves and b) Tafel plots for OER. c) Polarization curves and d) Tafel plots for HER. e) EIS Nyquist plots and f) double‐layer capacitance (*C*_dl_), in which I/II/III/IV/V represent Co‐ALMO@NF, ALMO@NF, Ni foams, IrO_2_, and Pt/C, respectively.

The multilayer microflower architecture of Co‐ALMO@NF is also benefit to the fast mass transport within electrodes. The OER performance of as‐prepared electrocatalysts was tested in 1 m KOH electrolyte by a three‐electrode system. From the linear sweep voltammograms displayed in **Figure** **3**a, it is observed that the Co‐ALMO@NF electrode shows a noticeable improvement for OER compared with Ni foam and ALMO@NF, and even surpasses the IrO_2_ benchmark. To achieve current densities of 10 and 100 mA cm^−2^, Co‐ALMO@NF requires overpotentials of 148 and 284 mV, respectively, which are lower than other control electrocatalysts: 301 and 373 mV for ALMO@NF, 309 and 370 mV for Ni foam, 257 and 322 mV for IrO_2_. Notably, a large current density up to 600 mA cm^−2^ can be displayed at the overpotential of 341 mV for Co‐ALMO@NF, which is ≈4.5 times higher current density of the benchmark IrO_2_ and 6.1 times that of ALMO@NF, demonstrating surprising OER activity, the overpotential comparison required for different current densities can be visually observed in Figure 3c. In addition, the inherent OER kinetics of electrodes above were also measured by the Tafel slope (Figure 3b), Co‐ALMO@NF shows a smaller Tafel slope of 53.9 mV dec^−1^, compared with ALMO@NF (74.5 mV dec^−1^), Ni foam (81.7 mV dec^−1^), and IrO_2_ (77.8 mV dec^−1^), reflecting superb electron transfer during the OER process. The HER performance of the electrocatalysts were also characterized under the same environment and Pt/C electrode was used as a benchmark for comparison. As shown in Figure 3c, Co‐ALMO@NF exhibits the overpotential of 159 and 299 mV to deliver the current density of 10 and 100 mA cm^−2^, while the overpotentials of ALMO@NF and Ni foam are 115/312 and 209/378 mV, respectively. Although the overpotential required for Pt/C electrode to deliver the current density of 10 mA cm^−2^ is only 68 mV, its overpotential rises sharply as the current density increases, the required overpotential for Pt/C to reach the current density of 100 mA cm^−2^ (302 mV) is exceeds that for Co‐ALMO@NF. Moreover, at a large current density of 500 mA cm^−2^, the overpotential required for Co‐ALMO@NF is only 349 mV. The smaller Tafel slope of Co‐ALMO@NF (101.4 mV dec^−1^) than ALMO@NF (179.1 mV dec^−1^) and Ni foam (174.9 mV dec^−1^) manifests the favorable HER kinetics of Co‐ALMO@NF (Figure 3d). In addition, the effect of Co doping on catalytic performance has been explored. Both the HER and OER performance of Co‐ALMO were enhanced at a Co salt addition of 112 mg (Figure S6, Supporting Information), when the molar ratio of Co/La/Mo was 0.61/1/3.83 (Table S1, Supporting Information). The catalytic kinetics was also analyzed by electrochemical impedance spectroscopy (EIS) to understand internal resistance and charge transfer resistance. A smaller semicircle of Co‐ALMO@NF from the Nyquist plot in Figure 3e displays lower charge transfer resistance (*R*_ct_), indicating an improved charge transport and reaction kinetics. In Figure 3f, the double‐layer capacitance (*C*_dl_) of the prepared electrocatalysts were obtained in non‐Faradaic region to evaluate the electrochemical surface areas (ECSA), corresponding cyclic voltammetry (CV) curves were shown in Figure S7 in the Supporting Information. Co‐ALMO@NF displays the highest *C*_dl_ value of 18.3 mF cm^−2^, which is 2.1‐ and 9.6‐fold times higher than those of ALMO and Ni foam, respectively, indicating that Co‐ALMO@NF exhibits more effective exposure of ECSA. The Co‐ALMO@NF performed better than the vast majority of listed non‐noble metal electrocatalysts (Table S3, Supporting Information).

**Figure 4:**
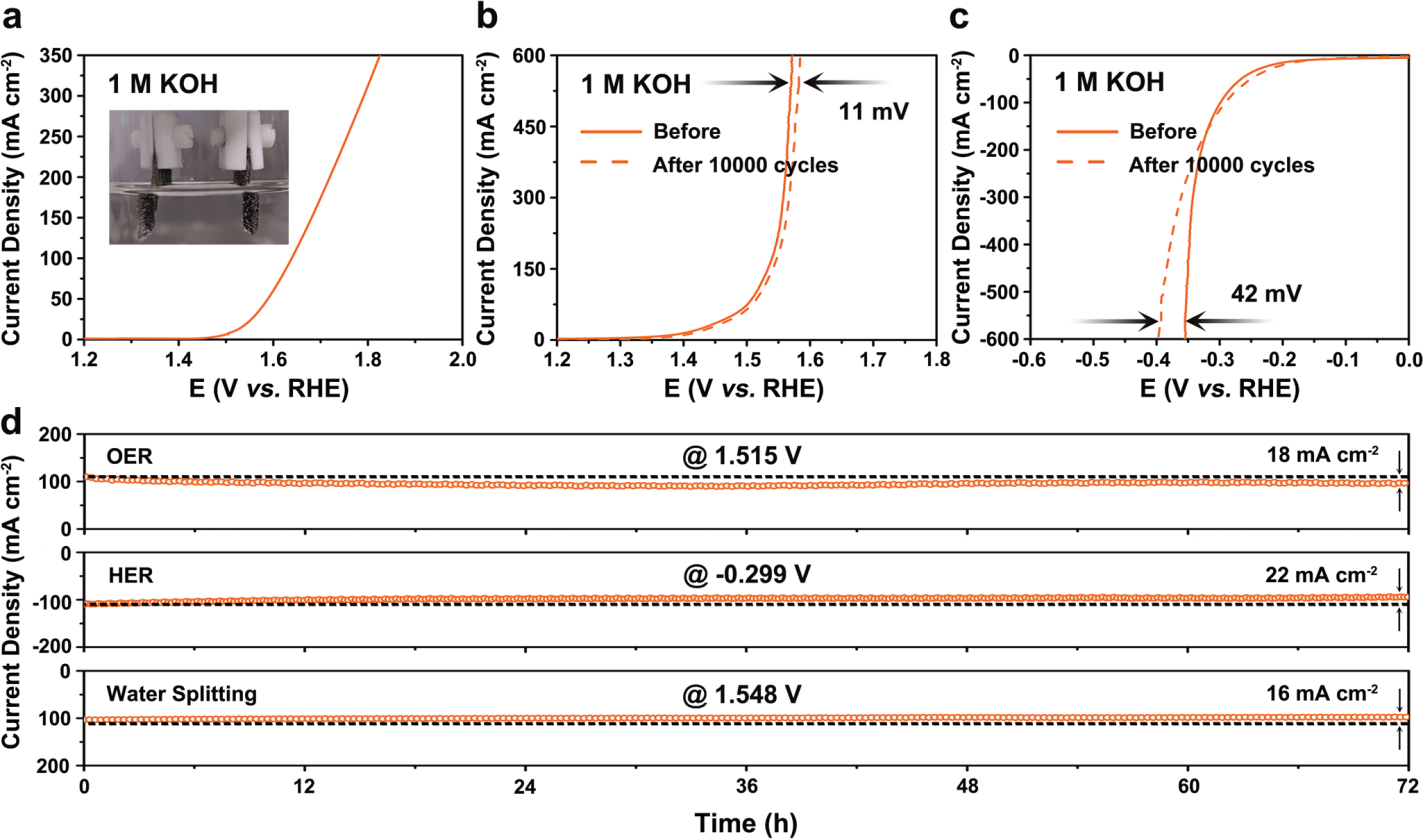
a) Polarization curve and the photograph of an electrolytic cell using Co‐ALMO@NF as both the cathode and anode for overall water splitting. Polarization curves of Co‐ALMO@NF (I) before and after 10 000 cycles for b) OER and c) HER. d) Chronoamperometric profile under different potentials of Co‐ALMO@NF.

Encouraged by the performance above, a two‐electrode system (Co‐ALMO@NF || Co‐ALMO@NF) was set up for overall water splitting in which the Co‐ALMO@NF electrodes as both the anode and cathode (inset of **Figure** **4**a). The polarization curve in Figure 4a reveals an excellent activity of as‐assembled device that only requires the voltage of 1.52 and 1.63 V at 10 and 100 mA cm^−2^, respectively. Notably, Co‐ALMO@NF as a core actuates the current density to 350 mA cm^−2^ at a low overall voltage of 1.82 V. Remarkably, a two‐electrode cell assembled by Co‐ALMO@NF presents long‐term stability by CV scanning and chronoamperometry. The polarization curves of Co‐ALMO@NF show slight changes after 10 000 cycles for both OER and HER (Figure 4b,c). In contrast, ALMO@NF and NF show a significant increase in overpotential after 10 000 cycles of CV (Figure S8, Supporting Information). Notably, there is no obvious attenuation of the real‐time current density under different constant potentials to maintain 100 mA cm^−2^ over 70 h (Figure 4d), demonstrating the modified molybdate stabilized on the Ni foam as a promising candidate for overall water splitting.

Moreover, DFT‐based first‐principles calculations were performed to reveal the reaction mechanism of HER and OER over the surface of Co‐ALMO electrocatalyst. The model of ALMO (010) facet is built (Figure S9, Supporting Information). As shown in Figure S10a in the Supporting Information, the charge surrounding Mo atom is decreased due to the introduction of Co. For investigating catalytic process, Co‐ALMO (010) model is built through replacing La by Co atom (Figure S11a, Supporting Information). Free energy diagram for OER is shown in Figure S10b in the Supporting Information, where the rate‐determining step is *O → *OOH. It also suggests that the adsorption strength on Co‐ALMO is too strong, that is, a less strong adsorption is favorable for OER. The energy diagram for HER is shown in Figure S10c in the Supporting Information, which indicates Mo is the most favorable active site for HER. The optimized geometries of hydrogen adsorption are provided in Figure S12 in the Supporting Information. A comparative plot of projected density of states (Figure S10d, Supporting Information) suggests that the doped Co can make d‐band center negatively shift, which will decrease the adsorption strength. It is in accordance with our experimental observation that Codoping can enhance the activity for both OER and HER, which is enabled by weakened adsorption after Codoping.

## 3 Conclusion

In summary, Co‐ALMO@NF with flower‐like microstructures were grown on conductive Ni foams by a facile one‐step hydrothermal reaction. It was proved to be cost‐effective bifunctional electrocatalysts for water splitting. Based on an enhanced electrochemical surface area and rich active species for water splitting, bifunctional Co‐ALMO@NF electrocatalysts possess superior activities in an alkaline media with low overpotentials of 159 and 148 mV to achieve 10 mA cm^−2^, 351 and 341 mV to achieve 600 mA cm^−2^ for HER and OER respectively. The cell voltage of two‐electrode electrolyzer fabricated by employing Co‐ALMO@NF as both the cathode and the anode is only 1.52 and 1.64 V to drive the current density of 10 and 100 mA cm^−2^, respectively. Furthermore, the DFT results indicate that the Mo site and the doped Co are specific active sites for HER and OER, respectively. The active sites and the electronic conductivity are stimulated by constructing lattice defects, which can reinforce the mutual effect from electronic structures and then expedite the charge transfer in the catalyst structure, and giving rise to better electrocatalytic performances. Considering the excellent catalytic performance, as well as the low‐cost and facile synthesis, the Co‐ALMO@NF can hold a great promise as a sustainable electrode material toward industrially applicable water splitting for hydrogen generation.

## Conflict of Interest

The authors declare no conflict of interest.

## Supporting information

Supporting Information

## Data Availability

The data that support the findings of this study are available from the corresponding author upon reasonable request.
