# Supplementary material for: Multilayered Molybdate Microflowers Fabricated by One‐Pot Reaction for Efficient Water Splitting
Source: Adv Sci (Weinh). 2023 Mar 22;10(14):2206952. doi: 10.1002/advs.202206952 (PMC10190612; doi:10.1002/advs.202206952)
Supplement: Supplementary file 1 — Supporting Information [file ADVS-10-2206952-s001.pdf]

Supplementary Information for

Multi-layered molybdate micro-flowers fabricated by one-pot reaction for efficient water splitting

1. Experimental Details

Synthesis of ALMO@NF: All the chemicals were directly used after purchase without further purification. 69.6 mg of fumaric acid and 288 mg (4.79 mmol) of urea were dissolved in 10 mL of deionized water at room temperature (solution A), 346 mg (0.28 mmol) of ammonium molybdate were dissolved in 15 mL of deionized water (solution B), 86 mg (0.20 mmol) of lanthanum nitrate was dissolved in 5 mL of deionized water (solution C). Then, solution A, solution B and solution C were mixed *via* stirring at room temperature (solution D). The solution D was transferred into a 50 mL Teflon-lined stainless autoclave fixed with one piece of commercial Ni foam (2 × 4 cm^2) at the bottom and control temperature rise and drop through the program maintaining at 90°C for 6 h. The sample was then removed from the solution, washed several times with DI water, and dried at 60°C for 1 day prior to electrochemical testing.

Synthesis of Co-ALMO@NF-x: All the chemicals were directly used after purchase without further purification. 69.6 mg of fumaric acid and 288 mg (4.79 mmol) of urea were dissolved in 10 mL of deionized water at room temperature (solution A), 346 mg (0.28 mmol) of ammonium paramolybdate and x mg of cobaltous acetate [x = 56/112/224/448 (0.32/0.64/1.28/2.56 mmol), where not otherwise stated in the text, x = 112] were dissolved in 15 mL of deionized water (solution B), and 86 mg (0.20 mmol) of lanthanum nitrate was dissolved in 5 mL of deionized water (solution C). Then, solution A, solution B and solution C were mixed via stirring at room temperature (solution D). The solution D was transferred into a 50 mL Teflon-lined stainless autoclave fixed with one piece of commercial Ni foam (2 × 4 cm^2) at the bottom and control temperature rise and drop through the program maintaining at 90°C for 6 h. The sample was then removed from the solution, washed several times with DI water, and dried at 60 °C for 1 day prior to electrochemical testing.

Characterization

The microstructures and compositions of samples were analyzed using scanning electron microscopy (SEM, Zeiss Merlin Compact), transmission electron microscopy and high-resolution transmission electron microscopy (TEM and HRTEM, FEI TalosF200S). TEM specimen was dispersed in ethanol and sonicated for 2 h before drop casting on a copper grid. XRD patterns were recorded using a Bruker D8 Advance X-ray diffractometer with a Cu Kα source. The 2θ angular region of the XRD pattern between 5° and 70° was studied at a scan rate of 0.1 s^{-1} with each step of 0.02°. X-ray photoelectron spectroscopy (XPS) measurements were performed on an ESCALAB 250 instrument (Thermo Electron) with Al Kα radiation. The specific surface area of the sample was evaluated by Brunauer-Emmett-Teller (BET) method, and the nitrogen (N_2) adsorption–desorption isotherms were measured on an adsorption apparatus (ASAP 2460 Micromeritics instrument) at 77 K.

Elemental content (Co, Mo and La) in Co-ALMO was determined by ICPAES (Optima 8000).

Electrochemistry Measurement

Electrochemical measurements were carried out in a typical three-electrode glass cell connected to a CHI 660E Electrochemical Workstation (CHI Instruments, Shanghai Chenhua Instrument Corp., China). For the hydrogen evolution reaction (HER) and oxygen evolution reaction (OER) tests, we used a standard three-electrode system with the prepared samples serving as the working electrode, a graphite rod serving as the counter electrode, and a standard Ag/AgCl electrode serving as the reference. 1 M KOH was used as the electrolyte, and the pH was about 14. OER polarization curves were obtained at a scan rate of 5 mV s^{-1} and stability tests were conducted under a constant voltage. The ECSA was measured by cyclic voltammetry (CV) using the same working electrodes at a potential window of -0.18~-0.08 V *vs.* Ag/AgCl in electrolytes. CV curves were obtained at different scan rates of 10, 20, 30, 40, 50, 60, 70, 80, 90 and 100 mV s^{-1}. After plotting charging current density differences ($\Delta J = J_a - J_c$ at the potential of -0.13 V *vs.* Ag/AgCl) versus the scan rates, the slope, twice of the double-layer capacitance C_{dl}, is used to represent the ECSA. Electrochemical impedance spectra (EIS) were obtained at open circuit voltage from 0.1 Hz to 100 KHz with an amplitude of 5 mV.

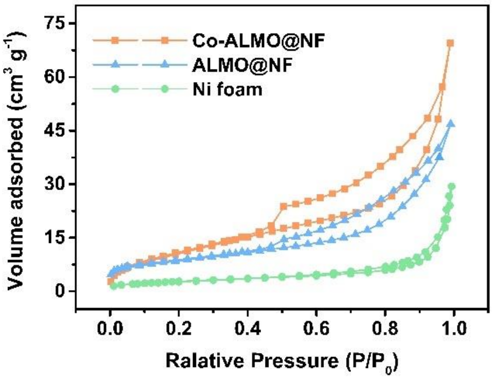

Figure S1. N_2-adsorption/desorption isotherms of Co-ALMO@NF, ALMO@NF and Ni foam.

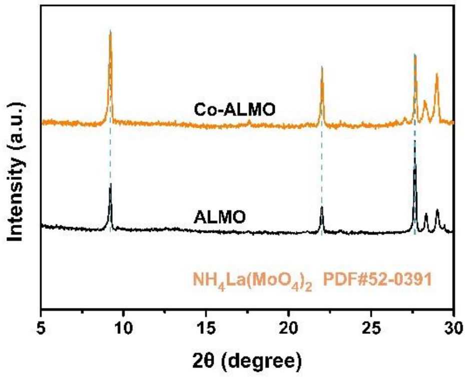

Figure S2. XRD patterns of Co-ALMO and ALMO zoom-in regions.

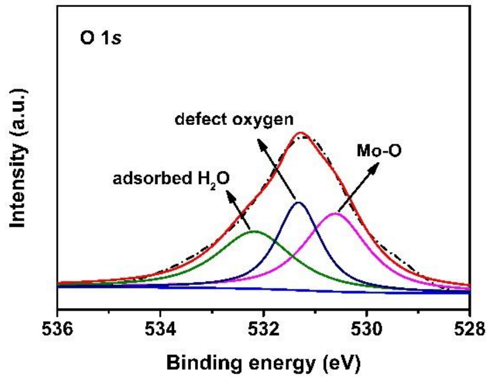

Figure S3. XPS analysis of O 1*s* for Co-ALMO.

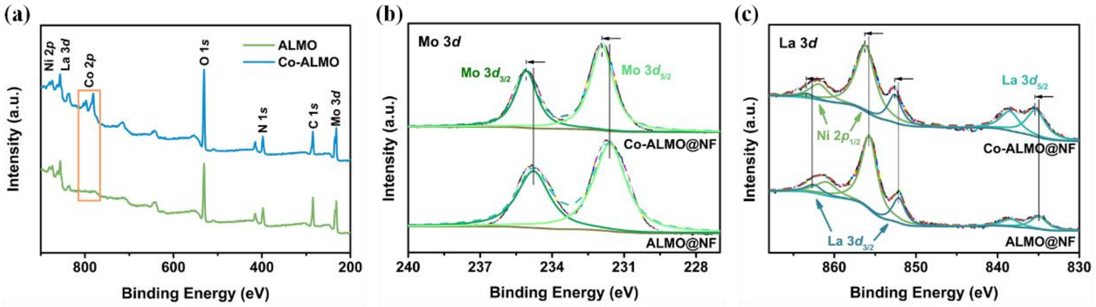

Figure S4. (a) XPS survey, (b) Mo 3*d* and (c) La 3*d* spectra for Co-ALMO@NF and ALMO@NF.

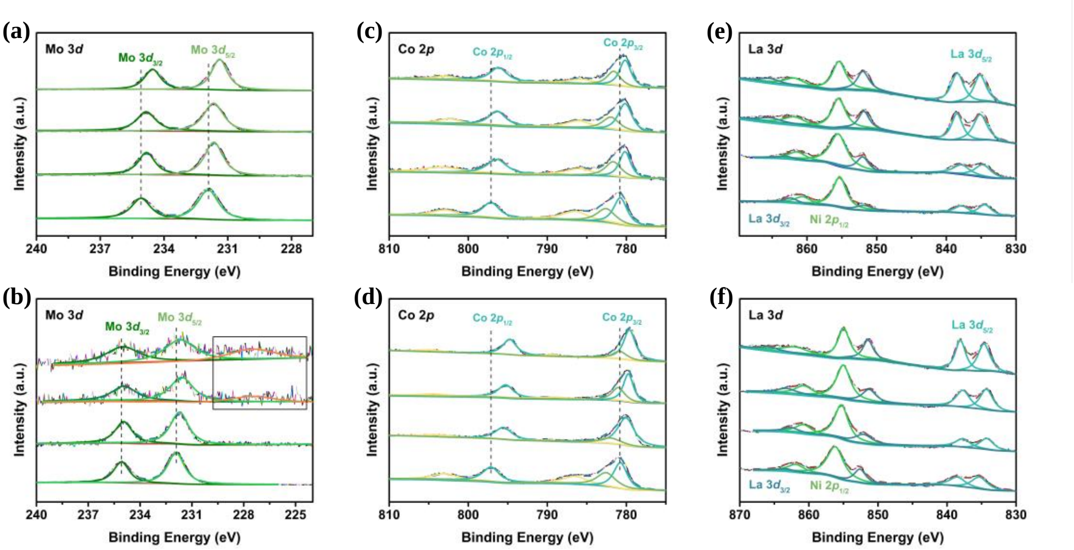

Figure S5. (a, b) Mo 3*d*, (c, d) Co 2*p* and (e, f) La 3*d* spectra for Co-ALMO@NF before and after CA tests. The four curves from bottom to top in (a, c e) are XPS spectra of Co-ALMO@NF after the CA tests at -0.299 V for 0, 30, 60, 90 min,

respectively; the four curves from bottom to top in (b, d f) are XPS spectra of Co-ALMO@NF after the CA tests at 1.514 V for 0, 30, 60, 90 min, respectively.

As shown above, Mo 3*d*, Co 2*p* and La 3*d* shifts to low binding energy while Ni 2*p* shifts to high binding energy during CA tests of both HER and OER, indicating that Mo, Co and La tends to lose electrons and conversely Ni tends to receive electrons; a new oxidation peak appears in Mo 3*d* spectrum during OER process (Figure S5b), which further supports the above conclusion.

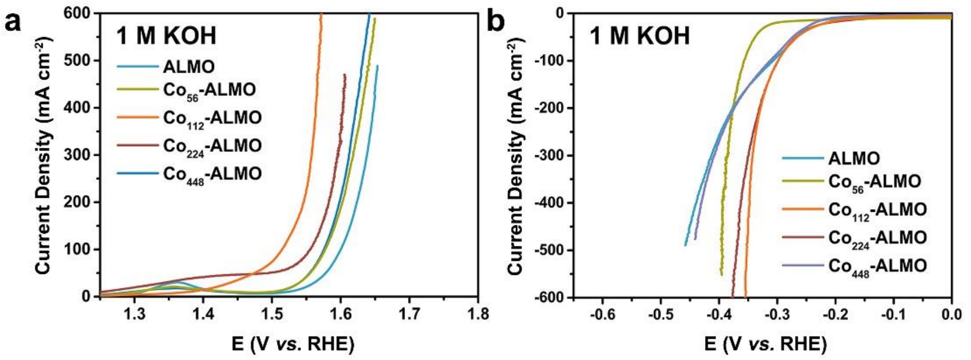

Figure S6. Polarization curves for (a) OER and for (b) HER of ALMO@NF, Co_{58}-ALMO@NF, Co_{112}-ALMO@NF, Co_{224}-ALMO@NF, Co_{448}-ALMO@NF.

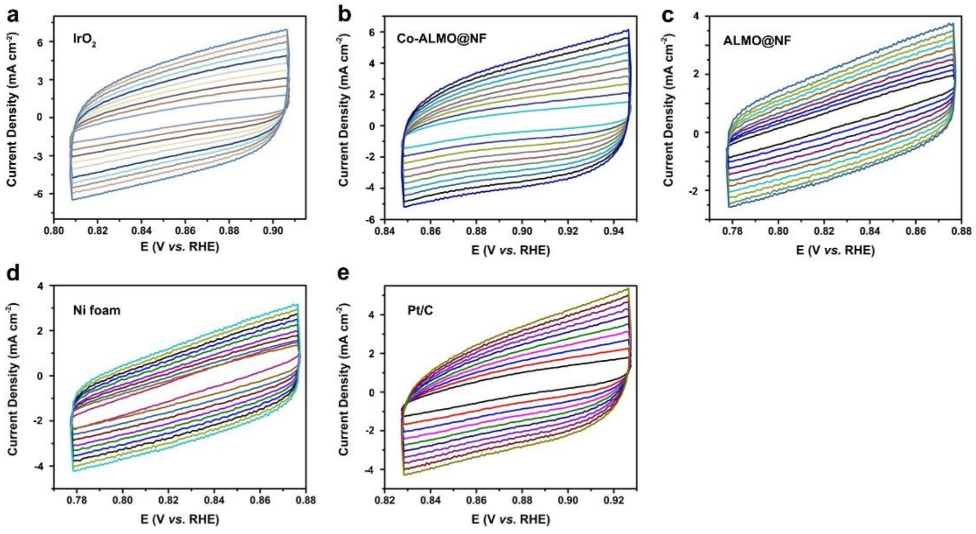

Figure S7. CV curves of (a) IrO_2, (b) Co-ALMO@NF, (c) ALMO@NF, (d) Ni foam and (e) Pt/C at scan rates ranging from 10 mV s^{-1} to 100 mV s^{-1} with an interval point of 10 mV s^{-1}.

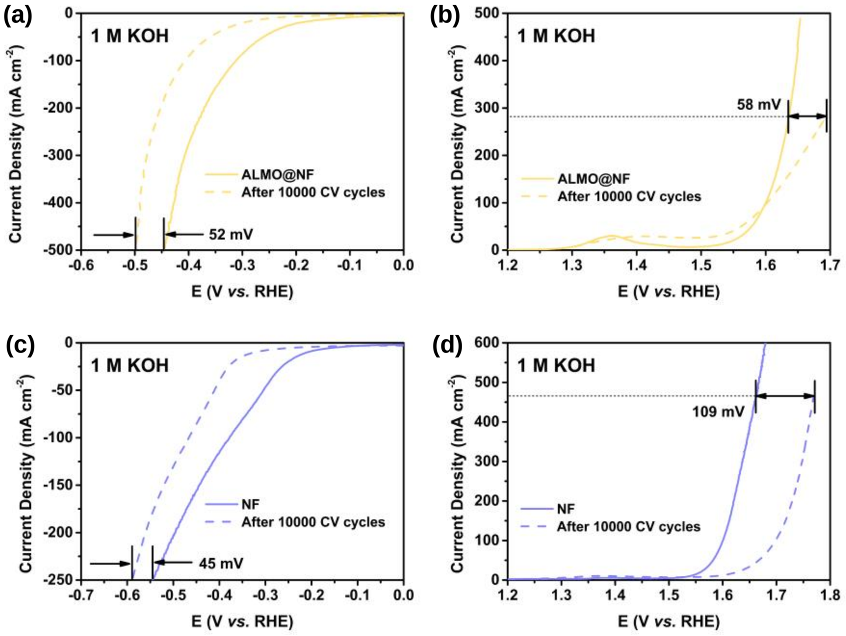

Figure S8. Polarization curves of ALMO@NF before and after 10,000 cycles for HER (a) and OER (b). Polarization curves of NF before and after 10,000 cycles for HER (c) and OER (d).

Table S1. Co doping amount of Co-ALMO@NF.

Catalyst	Element content (wt%)	Molar ratio
Co-ALMO-112	Co: 4.4<br>Mo: 44.23<br>La: 16.7	0.61: 1: 3.83

Table S2. Summary of the HER and OER activity of Ni foam, ALMO@NF and Co-ALMO@NF in 1.0 M KOH.

Catalysts (1 M KOH)	HER-η_{10} (mV)	HER-η_{100} (mV)	OER-η_{10} (mV)	OER-η_{100} (mV)
Ni foam	209	378	309	370
ALMO@NF	115	312	301	373
Co-ALMO@NF	159	299	148	284

Table S3. Comparisons of electrocatalytic performance for Co-ALMO@NF with other non-noble metal electrocatalysts in alkaline media.

Bifunctional electrocatalyst	Electrolyte	HER performance		OER performance		Overall water splitting	Reference
		η_{10} (mV)	Tafel slope (mV dec^{-1})	η_{10} (mV)	Tafel slope (mV dec^{-1})	η_{10} (mV)	
Co-ALMO@NF	1 M KOH	159	101	148	54	288	This work
Co_4S_3/Mo_2 C-N SC	1 M KOH	83	62	268	61	390	[1]
$Co_5Mo_{1.0}O$ NSs@NF	1 M KOH			270	54	450	[2]
$Co_5Mo_{1.0}P$ NSs@NF		173	190				
Co-Mo-P-O	1 M KOH	98	/	169	84	340	[3]
Co-P	1 M KOH	94	42	345	47	~410	[4]
MoP/Ni_2P/NF	1 M KOH	75	100	/	78	320	[5]
Mo_2C-MCNFs	1 M KOH	114	88	320	68	450	[6]
CoP/CNFs	1 M KOH	225	101	325	29	420	[7]
NiMo HNRs	1 M KOH	92	76	310	47	410	[8]
MoO_2 NS	1 M KOH	25	41	260	54	300	[9]
MoS_2/Ni_3S_2	1 M KOH	110	83	218	88	330	[10]
Co-P NS/C	1 M KOH	111	71	277	86	310	[11]
Co-P MNA	1 M KOH	54	51	290	65	390	[12]

2. Theoretical Section

2.1 Computational details

Density functional theory (DFT)-based calculations were performed on the platform of the Vienna ab initio simulation package (VASP) with the basis generated by projector augmented waves (PAW).[13] The function of Perdew, Burke and Ernzerhof (PBE)[14] was used for the description of the exchange and correlation potential. Monkhorst-Pack[15] based $3 \times 3 \times 1$ k points were sampled in the reciprocal zone. The criteria of convergence were set to 1×10^{-5} eV and 0.02 eV/Å for electronic and ionic steps, respectively. DFT-D3 method of Grimme[16] was adopted to describe vdW dispersion.

The model of Co-ALMO (010) surface was built *via* replacing the La atom by Co atom with subtracting the neighboring O atom. The model contains a vacuum layer of 15 Å for avoiding the

interaction between periodic images. Free energy diagrams of HER[17] and OER[18] were estimated by the methods referred by previous research, respectively. Free energy is calculated as the equation:

$\Delta G = \Delta E + T\Delta S + \Delta ZPE$

where ΔG, ΔE, ΔT, ΔS, and ΔZPE represents relative free energy, energy, vibration entropy, temperature and zero-point energy. $\Delta ZPE - T\Delta S$ = 0.24,[17] 0.05, 0.35, and 0.40 eV[18, 19] for ΔG_H, ΔG_O, ΔG_{OH}, and ΔG_{OOH}, respectively.

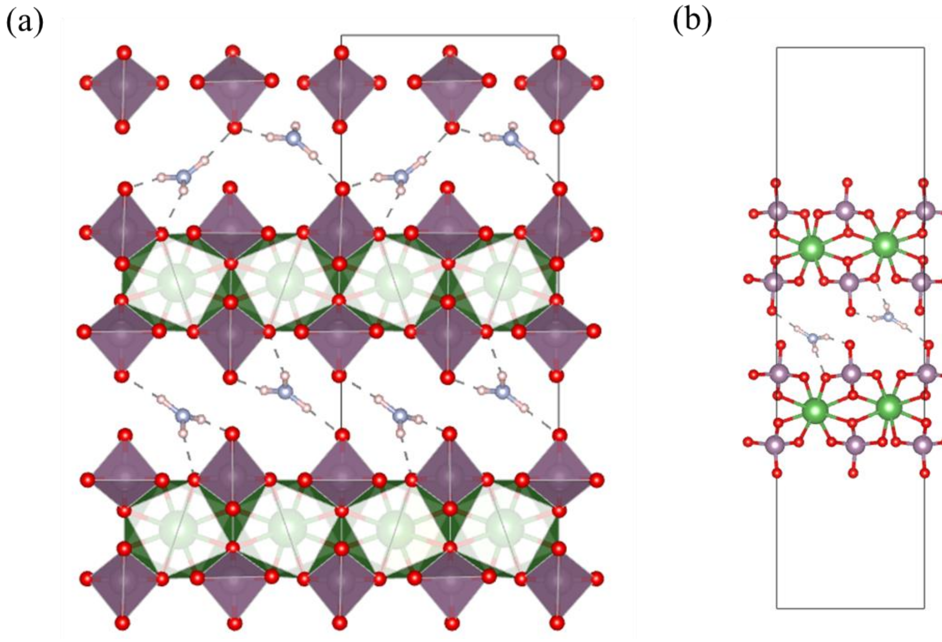

Figure S9. (a) The unit cell of ALMO. (b) (010) facet of ALMO.

2.2 Theoretical models

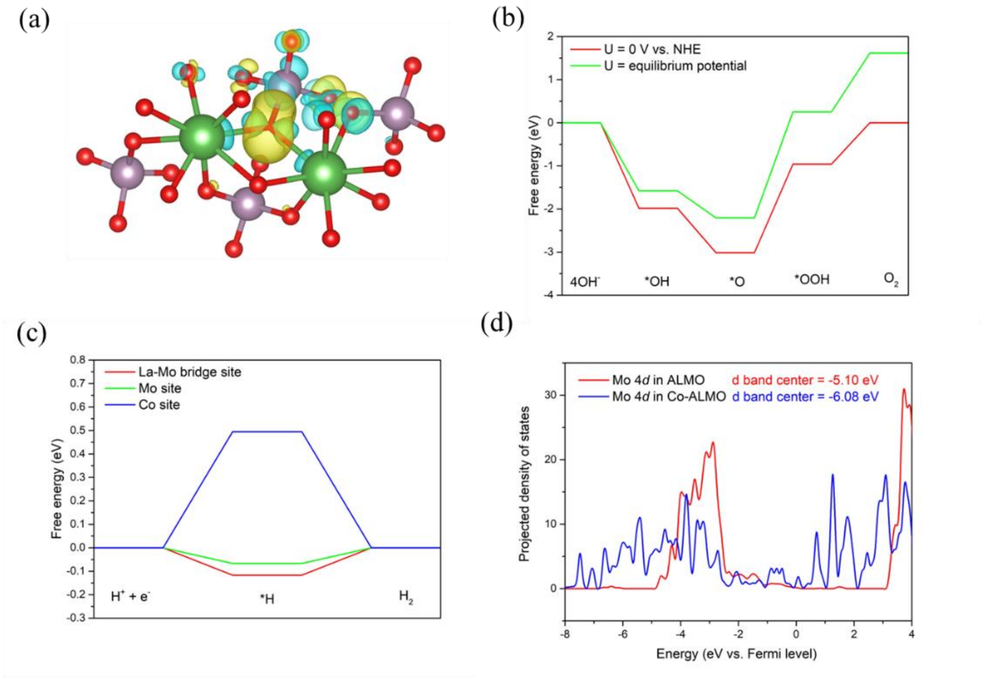

Figure S10. (a) Charge difference between perfect ALMO (010) surface and that with oxygen vacancy (isosurface: 0.005 e/bohr^3, yellow is increase and blue is depletion). (b) Free energy diagram of OER. (c) Free energy diagram of HER. (d) Projected density of states for ALMO and Co-ALMO.

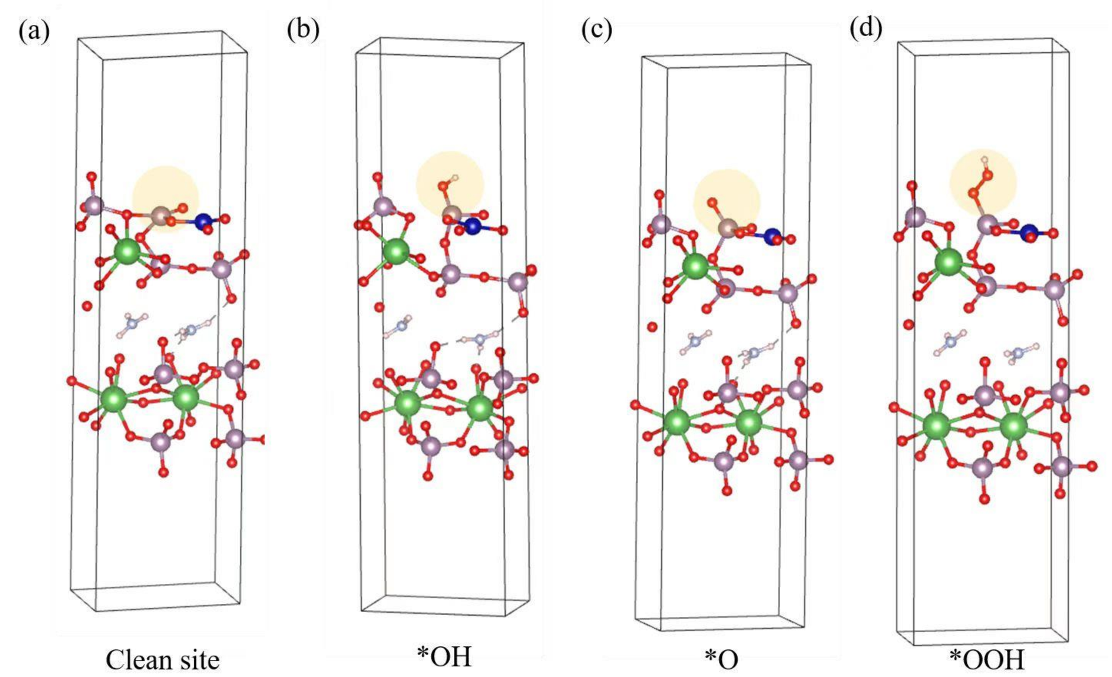

Figure S11. (a) The model of Co doped ALMO (010) facet. The optimized adsorption structure of (b) *OH, (c) *O and (d) *OOH. The adsorption species are denoted in light yellow circles.

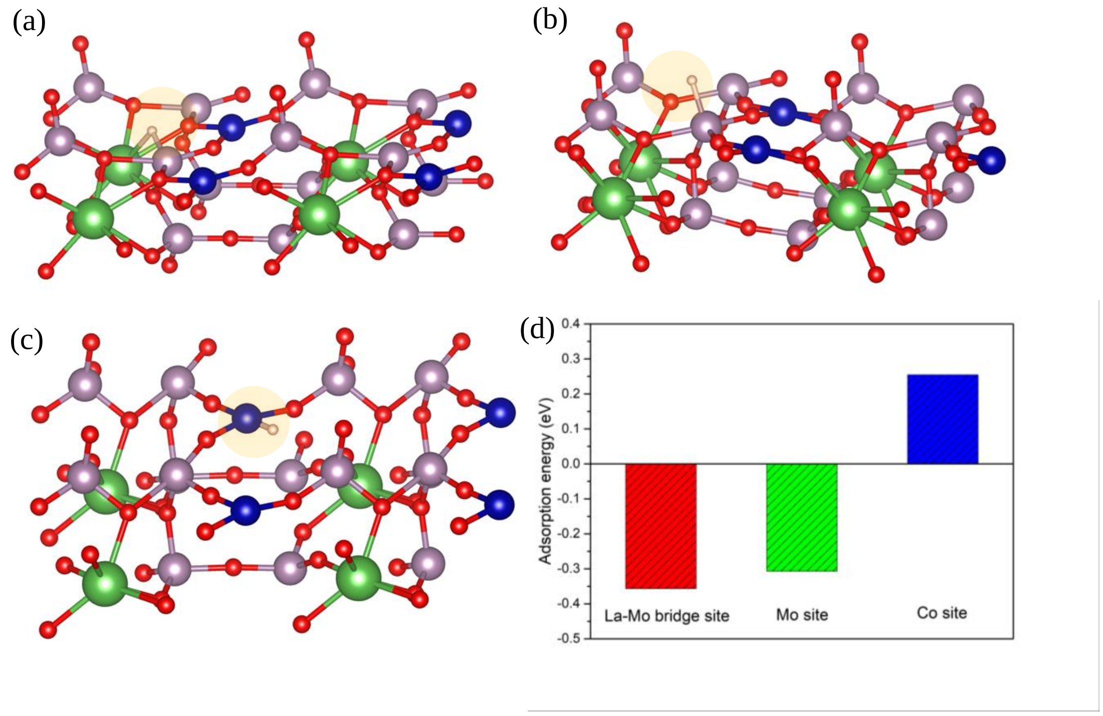

Figure S12. The optimized adsorption structure of *H on different active sites. (a) La-Mo bridge site. (b) Mo site. (c) Co site. Adsorption species are denoted in light yellow circles. (d) The adsorption energy diagram.

Reference:

[1] Y. Liu, X. Luo, C. Zhou, S. Du, D. Zhen, B. Chen, J. Li, Q. Wu, Y. Iru, D. Chen, Applied Catalysis B: Environmental 2020, 260, 118197.

[2] Y. Zhang, Q. Shao, S. Long, X. Huang, Nano Energy 2018, 45, 448.

[3] X. Wang, G. She, L. Mu, W. Shi, ACS Sustainable Chemistry & Engineering 2020, 8, 2835.

[4] N. Jiang, B. You, M. Sheng, Y. Sun, Angewandte Chemie 2015, 127, 6349.

[5] C. Du, M. Shang, J. Mao, W. Song, J. Mater. Chem. A 2017, 5, 15940.

[6] C. Ji, G. Yang, P. R. Ilango, J. Song, D. Yu, S. Han, D. Zhang, L. Li, S. Peng, Chemistry–An Asian Journal 2020, 15, 1957.

[7] X.-Q. Xie, J. Liu, C. Gu, J. Li, Y. Zhao, C.-S. Liu, Journal of Energy Chemistry 2022, 64, 503.

[8] J. Tian, N. Cheng, Q. Liu, X. Sun, Y. He, A. M. Asiri, J. Mater. Chem. A 2015, 3, 20056.

[9] Y. Jin, H. Wang, J. Li, X. Yue, Y. Han, P. K. Shen, Y. Cui, Advanced Materials 2016, 28, 3785.

[10] J. Zhang, T. Wang, D. Pohl, B. Rellinghaus, R. Dong, S. Liu, X. Zhuang, X. Feng, Angewandte Chemie 2016, 128, 6814.

[11] J. Chang, L. Liang, C. Li, M. Wang, J. Ge, C. Liu, W. Xing, Green Chemistry 2016, 18, 2287.

[12] Y. P. Zhu, Y. P. Liu, T. Z. Ren, Z. Y. Yuan, Advanced Functional Materials 2015, 25, 7337.

[13] P. E. Blöchl, Physical review B 1994, 50, 17953.

[14] J. P. Perdew, K. Burke, M. Ernzerhof, Physical review letters 1996, 77, 3865.

[15] H. J. Monkhorst, J. D. Pack, Physical review B 1976, 13, 5188.

[16] S. Grimme, S. Ehrlich, L. Goerigk, Journal of computational chemistry 2011, 32, 1456.

[17] J. K. Nørskov, T. Bligaard, A. Logadottir, J. Kitchin, J. G. Chen, S. Pandelov, U. Stimming, Journal of The Electrochemical Society 2005, 152, J23.

[18] J. K. Nørskov, J. Rossmeisl, A. Logadottir, L. Lindqvist, J. R. Kitchin, T. Bligaard, H. Jonsson, The Journal of Physical Chemistry B 2004, 108, 17886.

[19] J. Rossmeisl, Z.-W. Qu, H. Zhu, G.-J. Kroes, J. K. Nørskov, Journal of Electroanalytical Chemistry 2007, 607, 83.
